# Group Membership Trumps Shared Preference in Five-Year-Olds’ Resource Allocation, Social Preference, and Social Evaluation

**DOI:** 10.3389/fpsyg.2022.866966

**Published:** 2022-05-31

**Authors:** Li Yang, Youjeong Park

**Affiliations:** Department of Child Development and Family Studies, Seoul National University, Seoul, South Korea

**Keywords:** group membership, social liking, third-party evaluation, resource distribution, shared interest, five-year-olds

## Abstract

This study investigated five-year-olds’ priority between shared preference and group membership in resource allocation, social preference, and social evaluation. Using a forced-choice resource allocation task and a friend choice task, we first demonstrate that five-year-old children distribute more resources to and prefer a character who shares a preference with them when compared to a character who has a different preference. Then, we pitted the shared preference against group membership to investigate children’s priority. Children prioritized group membership over shared preference, allotting more resources to and showing more preference toward characters in the same group who did not share their preferences than those from a different group who shared their preferences. Lastly, children evaluated resource allocation and social preference in others that prioritized group membership or shared preference. Children regarded prioritization of group membership more positively than prioritization of shared preference from the perspective of a third person. The results suggest that children by five years of age consider group membership as of greater importance than shared preference not only in their own resource allocation and social preference, but also in their evaluation of others’ resource allocation and liking.

## Introduction

Children’s social behaviors are often based on choices. For example, choosing which friend to share candies or play with presents a child with multiple options; children may also select whom to help in some contexts (e.g., Dunfield and Kuhlmeier, 2010; Vaish et al., 2010; Kenward and Dahl, 2011; Dahl et al., 2013). A considerable amount of research has focused on identifying the bases of these selections, such as social group membership, reciprocity, and others’ past moral and immoral behavior (for review, see Kuhlmeier et al., 2014). Relatively little attention has been paid, however, to examining the bases that children consider more important than others while making such decisions, despite its relevance for predicting and understanding their behaviors.

One foundation of young children’s social liking is shared preferences. Children by two years recognize similarity in preferences between themselves and others (Fawcett and Markson, 2010a). At three years, they prefer to play with peers whose food and toy preferences match their own (Fawcett and Markson, 2010b). Further, four- to six-year-old children, most of whom have at least one focused interest which persists for some time (e.g., dinosaurs and constructive play; Alexander et al., 2008), report common play and interests as important criteria for friend selection (Rekalidou and Petrogiannis, 2012). In addition, experimental studies that presented a variety of interests such as books, games, and TV shows by way of character representations such as pictures of peers have revealed children’s liking for same-preference individuals at four to six years (Sparks et al., 2017) and six to nine years (Heiphetz et al., 2014).

Moreover, although few studies have investigated the effects of shared preferences on young children’s generosity in resource allocation, limited evidence suggests that shared preferences can influence young children’s resource distribution to some extent. When asked to distribute stickers between themselves and a fictional peer, four- to six-year-old children distributed less to peers who disliked their preferences than to those who shared their preferences or whose opinions were unknown (Sparks et al., 2017). In addition, three- to six-year-old children who had equally distributed resources to in-group and out-group members switched to unfair allocation favoring their in-group after being informed that their in-group, but not out-group, shared their preferences (Sudo, 2021). Although findings are limited to children from Western countries, these findings together with those regarding young children’s friend choice suggest that shared preference is closely linked to affiliation formation and an increased possibility of positive social behaviors through the preschool years.

Young children’s affiliation with others who share their preferences may be explained by the general preference for *similar others*, a preference evident from infancy (e.g., Mahajan and Wynn, 2012). According to Dishion et al. (1994), interpersonal similarity may lead to an emotional sense of connectedness that begins the process of becoming friends. The initial feeling of connectedness serves as a guide to discovering more similarities and establishing a friendship. Therefore, shared preferences cannot guarantee, but at least indicate a possibility of a good relationship. Young children’s generosity to those with similar preferences may also be explained by the feeling of affiliation (Sparks et al., 2017). In addition to the general effect (i.e., the positivity toward those who are like them), more strategic motives may partially contribute to children’s generosity to those who share their preferences. As Sparks et al. (2017) postulated, an interpersonal compatibility may make investing in a relationship seem more promising.

Another well-established factor of young children’s social liking is group membership—namely, whether the person belongs to the same group as the child (in-group) or not (out-group). Particularly, a social group is defined as two or more people who interact with one another, share similar characteristics, and collectively have a sense of unity (Reicher, 1982). Among various types of groups (Lickel et al., 2000), group membership in social groups such as social categories (e.g., nationality) and task groups (e.g., a team) has been shown to influence social liking. For example, three-year-olds show a stronger tendency toward friendship with their same-sex peers (Shutts et al., 2013). Five-year-old Caucasian children tended toward friendship with children who matched their race (Abel and Sahinkaya, 1962). In addition, five-year-old American children who spoke English wanted to be friends with English-speaking peers more than French-speaking peers and preferred those who spoke English in their native accent more than those who spoke with a French accent (Kinzler et al., 2009). Furthermore, in a minimal group task in which children were randomly assigned to groups (teams) by a temporary minimal criterion, five-year-olds rated their liking for in-group members higher than out-group members (Dunham et al., 2011; Sudo, 2021).

Group membership can also play a role in young children’s resource allocation. Three- to six-year-old children distributed more resources to in-group than out-group members in groups assigned according to gender (Dunham et al., 2011), race (Renno and Shutts, 2015), or a combination of accent and race (Spence and Imuta, 2020). In minimal group contexts, young children distributed resources in favor of their in-group members (Sparks et al., 2017) although the tendency failed to reach statistical significance in a few studies (Dunham et al., 2011; Plötner et al., 2015a; Sudo, 2021).

The effects of group membership on young children’s social preference and resource allocation may be explained by developing group-mindedness. It has been argued that human beings have developed a unique way of thinking called *group-mindedness*, reflecting the importance of the group to human survival (Tomasello et al., 2012; Tomasello, 2019). Human beings live in groups, allowing individuals to protect themselves from external threats better, share important information among group members, and more efficiently select a mate for reproduction (Ward and Webster, 2016). Therefore, humans recognize group division, are more favorable to in-group than out-group members, and they pay attention to and abide by group norms, enabling the group to operate smoothly (Tomasello, 2019). This group-mindedness is evident from about three years of age (for review, see Tomasello, 2019). Young children begin to classify people into social groups based on appearance and behavior, and distinguish between in-group and out-group (e.g., Nesdale et al., 2004). They exhibit In-Group Favoritism (IGF; Nesdale et al., 2004; Patterson and Bigler, 2006; Shutts et al., 2013). In addition, children from the age of three will reprimand and correct an in-group member who breaks a social norm in a way that threatens the group’s function, and strive to bring the group back into line (Schmidt et al., 2012). Thus, although group-mindedness persists into adulthood (Billig and Tajfel, 1973; Brewer and Silver, 1978; Dobbs and Crano, 2001; Falk et al., 2014), the preschool period is crucial for its development.

While studies have demonstrated that shared preference and group membership impact preschool-aged children’s resource allocation and social preference, little research has examined which of the two factors more strongly affects such behaviors. In practice, members of the same social group may tend to share preferences (Reicher, 1982), but preferences may not always vary with group membership. That is, children may encounter in-group members with different preferences and out-group members with the same, which implies that shared preference and group membership can be in conflict with one another. Considering such conflict, Sudo (2021) examined how the conflicting versus non-conflicting cues about group membership and shared preferences would affect three- to six-year-old children’s social preference and resource allocation. Children who received the non-conflicting cues that their group (but not the out-group) shared their preferences rated their liking (measured on a five-point scale) and distributed resources in favor of their in-group. However, children who heard the conflicting cues that their out-group (but not the in-group) shared their preferences equally liked the groups. They also allocated resources equally to their in-group and out-group characters, just like they did before they were given the information about preferences. Together, the out-group was not favored over the in-group by these children despite its common preference, suggesting a limited impact of shared preference and a robustness of intergroup biases in the face of its conflict with shared preference.

The findings by Sudo (2021) have provided important initial evidence for the robustness of intergroup biases relative to the effect of shared preferences in young children’s social liking and resource allocation. Yet, the study had a limitation in revealing children’s priority between shared preference and group membership as bases of liking and favorable behaviors. Specifically, the task in Sudo’s (2021) study included not only the shared preference and group membership but also the equality rule as the choices; children always had the option of distributing resources equally (they were given eight coins and eight potential recipients, four of whom were in-group members), thus allowing young children’s preference for equal distributions of resources between individuals (e.g., LoBue et al., 2011; Cooley and Killen, 2015) to overshadow the choices between the shared preference and group membership. Possibly, young children’s priority was not clearly evident in Sudo’s (2021) study due to the equality option. This possibility calls for empirical research that excludes the equality option and focuses on demonstrating which of the shared preference and group membership children consider more important in *selective* favorable behaviors.

A combination of several research findings predicts that young children—especially, five-year-olds—would privilege group membership over shared preference in selective resource distribution and liking. First, Sudo (2021) measured children’s liking with two types of tasks (i.e., a five-point scale versus a forced-choice task in which they were asked to choose one that they liked more). Children of three to six years, when given the conflicting cues, trended toward preferring their in-group member with a different preference over an out-group member with a shared preference on the forced-choice task (*p* = 0.06). Second, evidence indicates that group loyalty becomes a strong factor in children’s own behaviors and evaluation of others’ between the ages of four and five years. Specifically, five-year-olds consider group loyalty so important that they willingly pay a personal cost for the benefit of their group even with minimal groups (Misch et al., 2016), consider a lie told in favor of in-group members more morally acceptable than for out-group (Jin et al., 2019), and are less likely to tattle on transgression of their group members when much is at stake for the group (Misch et al., 2018). Thus, moral reactions of five-year-olds are moderated by group loyalty in some contexts. In addition, children by five years expect group loyalty as a norm, evaluating loyal individuals positively but disloyal individuals negatively (Misch et al., 2014). These findings align well with the developing group-mindedness during the preschool years (Tomasello, 2019) and social identity development theory (Nesdale, 2004) that characterizes preschool-aged children with a focus on, and concern for, belonging to their group and positively distinguishing their in-group from out-groups. Taken together, it would be reasonable to expect that group membership would outweigh the general positivity effect of shared preference at least in older preschoolers’ resource allocation and social liking in forced-choice contexts.

Another question that remains unanswered concerns young children’s reasoning about group membership (and shared preference) as a basis of liking and favorable behaviors. In particular, children’s own choices in resource allocation and social liking do not speak to whether they have a normative sense that prioritizing one factor (e.g., group membership) over the other is something good and more appropriate, or their priority is merely behavioral inclinations. Children’s evaluation of others may provide a venue for exploring this question. If children have a normative sense that prioritizing group membership over shared preferences is desirable, then children should evaluate a character prioritizing group membership over shared preference positively, but a character showing the reverse priority negatively. Importantly, exploring this possibility requires one to test children’s evaluation of resource allocation and preference of others who are in *no* relation to the children, to rule out the possibility that the responses are based simply on the behaviors’ outcomes (e.g., more resources) to or their positive feelings for their own groups (Abrams et al., 2003) rather than based on a general, abstract understanding of the prioritization. This third-party evaluation of others’ resource allocation and preference in fact is likely to occur in young children’s lives. Preschool-aged children have rich opportunities to observe others’ resource allocation and preferences as they expand their scope of social experiences by attending preschools. They also spontaneously evaluate others’ behaviors that are not directed to them, based on the diverse social norms that they have acquired, such as fair allocation of resources and respect of others’ property right, as an unaffected bystander (e.g., Rossano et al., 2011; DeJesus et al., 2014; Hardecker et al., 2016; Hardecker and Tomasello, 2016). Thus, how children evaluate others’ resource allocation and preferences based on shared preference and group membership is a matter of interest. To our knowledge, however, no study has yet investigated the issue, particularly when shared preference and group membership are in conflict. Thus, it would be interesting to explore how children reason about peers who prioritize either group membership or shared preference in friend choice and resource allocation in forced-choice scenarios. This would be informative for theorizing on the development of a norm for prioritizing group membership over shared preference.

Although not a direct test of how children evaluate others who prioritize shared preference over group membership and those who show the reverse priority, an investigation of children’s evaluation of others’ group loyalty suggests the possibility that a normative stance toward group membership priority would be evident later in the preschool years. It is not until the age of five years that children not only choose group loyalty themselves (Misch et al., 2016, 2018) but also clearly regard group loyalty as being morally good and disloyal group members as being morally bad (“a betrayal”) in a third-party standpoint (Misch et al., 2014). Five-year-olds, who have demonstrated that loyal behavior is the expected norm (Misch et al., 2014), would negatively evaluate others’ behaviors that are more beneficial to out-group members than in-group members. Thus, it was predicted that five-year-old children would evaluate others’ resource allocation and friend choice that prioritized group membership more positively than those choices that prioritized shared preferences.

Based on the research gaps mentioned above, this study had three goals. The first was to test whether shared preference affects resource allocation and social preference among young children in an East Asian country. The second goal was to test whether children would prioritize shared preference or group membership for resource allocation and social preference when the two are in conflict and they have to favor one person over the other. Furthermore, we aimed to examine whether the choice would generalize to children’s evaluation of the resource allocation and liking of others, to obtain an insight into young children’s developing normative sense about prioritizing between shared preference and group membership in their selective social behaviors. Together, this study would provide novel data regarding the relative impact of shared preference and group membership on young children’s resource allocation, social preference, and social evaluation.

To achieve the goals, we pitted the characters’ group membership against shared preferences. For the purpose of the present study, we used fictional classes as groups. Although groups can be formed on the basis of various criteria including shared preferences (e.g., a music club), prior work has shown that five- to six-year-old children’s spontaneous definition of a “group” is limited to classes in kindergartens (Plötner et al., 2015b). Classes also meet the definition of a social group (i.e., two or more people who interact with one another, share similar characteristics, and collectively have a sense of unity; Reicher, 1982). Furthermore, our pilot study revealed that the term “class (班)” was familiar to and normally used by preschool-aged children in China, whereas the term “team (组 or 队)” was unfamiliar to them. We also reasoned that having to choose a play partner between a classmate with a dissimilar preference and a peer who has similar tastes from a different class does happen in young children’s lives. Likewise, conditions where children are required to benefit one person more than the other in resource distribution can occur for reasons such as limited resources. We expected that while shared preference would have an impact on young children’s resource allocation and social liking, five-year-old children would provide clear evidence for their prioritizing group membership over shared preference when they were to select one over the other as a play partner or one who benefits more. Moreover, their priority to group membership was predicted to generalize to their evaluation of others’ selective social liking and resource distribution.

## Materials and Methods

### Participants

Sixty-four five-year-old children (32 boys, 32 girls; *M* = 64.98 months, *SD* = 3.52 months) residing in Zhangjiakou City, Hebei Province, China, participated in this study. Four additional children participated but were excluded for the following reasons: two children’s answers were not recorded due to an equipment error; one child could not concentrate on the study due to a change in test place in the middle of test session; and one child did not understand the researcher’s explanation as indicated by the comprehension check. Among the participants, 14.06% (*n* = 9) were first born, 48.44% (*n* = 31) were second born, 7.81% (*n* = 5) were third born, and 29.69% (*n* = 19) were the only child. With regard to years of kindergarten attendance, two to three years was the most common (50.00%, *n* = 32), followed by three to four years (28.13%, *n* = 18), one to two years (20.31%, *n* = 13), and less than one year (1.56%, *n* = 1). Prior to data collection, we conducted an *a priori* analysis to determine a sample size required to detect an effect of medium size (0.5) in one-sample *t*-tests and paired *t*-tests. Given the alpha level of 0.05, a medium effect size of 0.5, and two-tailed tests, a required sample size was 54. The medium effect size was based on previous results. For example, in Sparks et al. (2017), the effect of shared preference on resource allocation was *d* = 0.47. Also, sample sizes of previous studies were 32 (Sparks et al., 2017, Exp.1 and Exp. 2), 81 six to nine year olds (Heiphetz et al., 2014), and 76 three to six year olds (Sudo, 2021, with one between-participants manipulation).

### Materials

#### Task 1: Resource Allocation and Social Preference Task Based on Shared Preference

**Figure 1:**
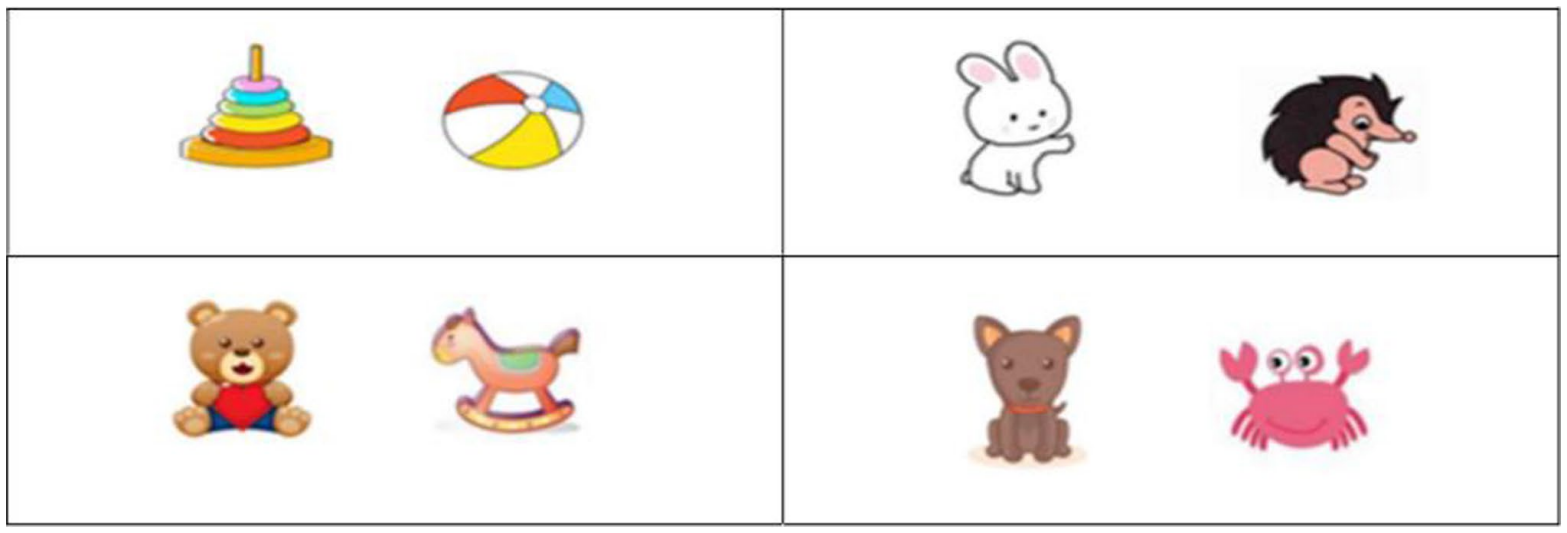
Four pairs of objects of preference used in Tasks 1 and 2. The images of toys and animals used in task 1 and 2 were obtained from the following free image websites (www.huihua8.com; www.jianbihua.com; www.51yuansu.com; www.baiqi008.com; www.jbhdq.com; www.photophoto.cn).

**Figure 2:**
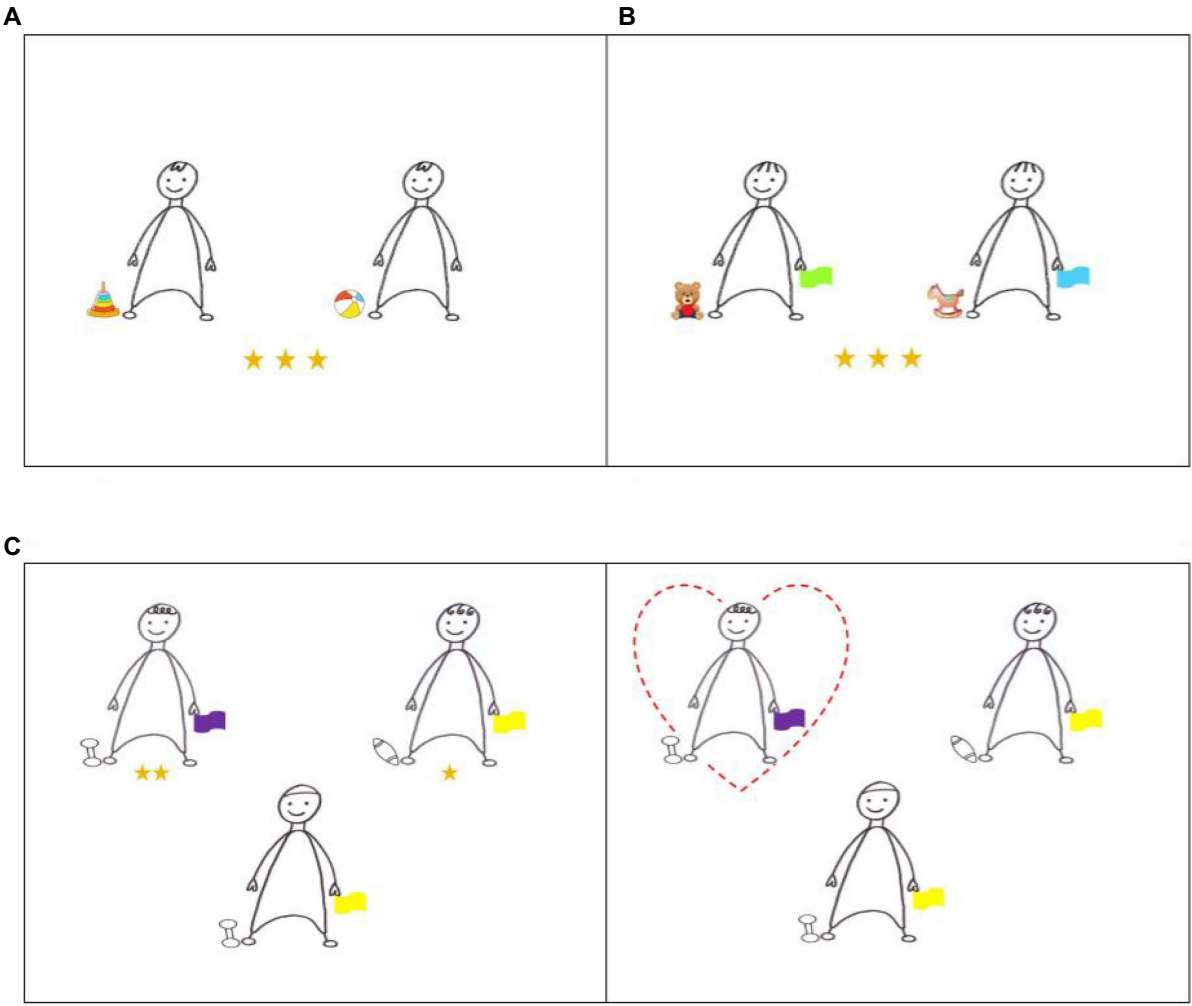
Sample items of Tasks 1 **(A)**, 2 **(B)**, and 3 **(C)**. The images of toys and animals used in task 1 and 2 were obtained from the following free image websites (www.huihua8.com; www.jianbihua.com; www.51yuansu.com; www.baiqi008.com; www.jbhdq.com; www.photophoto.cn).

The first task was designed to test children’s resource allocation and social preference based on shared preferences. Four pairs of toys or pets familiar to young Chinese children were selected as the objects of preference (Figure 1). Also, four picture cards of black and white line drawings, each showing a pair of characters that looked identical (Figure 2A), were constructed. The characters’ hairstyles varied with cards. One character in each pair shared a preference with the child, and the other had a different preference. Identical characters were used to control for the influence of facial expression, gender, and appearance on responses. Laminated pictures of the objects were used to demonstrate the characters’ preferences.

In addition, three stickers were used as resources for allocation as they are considered valuable to young children, are often used as rewards in kindergarten (Park et al., 2019), and have frequently been used in studies examining resource allocation in children (e.g., Cha and Song, 2015; Plötner et al., 2015a). We limited the number of resources to three, as young children’s ability to count has been shown to affect their distributive behavior (Chernyak et al., 2016; Choe et al., 2021), as exemplified in previous studies (Olson and Spelke, 2008; Shutts et al., 2013; Renno and Shutts, 2015). We measured children’s social preference using the friend selection method, which has been used widely in previous studies (Kinzler et al., 2009; Fawcett and Markson, 2010b; Shutts et al., 2013).

#### Task 2: Resource Allocation and Social Preference Task Based on Shared Preference vs. Group Membership

The second task aimed to determine children’s priorities in resource allocation and preference when shared preference was in conflict with group membership. The task consisted of four picture cards identical to those in Task 1, except that the characters’ group membership information was added. Group membership was indicated by the color of a flag held by the characters. Sky blue and light green were selected because they had neither positive nor negative meanings associated with them in Chinese culture. One character in each pair shared a preference with the child but belonged to a different group, whereas the other belonged to the child’s group but showed a preference for a different object. Again, children were asked to distribute three stickers between the two characters and choose a character they would like to be friends with Figure 2B.

#### Task 3: A Task of Third-Party Evaluation on Others’ Resource Allocation and Social Preference

The third task was constructed to test children’s evaluation of others’ resource allocation and preference that prioritized either shared preference or group membership. It consisted of eight picture cards showing three characters, two in the upper row and the third, the protagonist, at the bottom (Figure 2C). The task employed a new set of characters to avoid a carry-over impression from Tasks 1 and 2. The two upper characters shared either preference or group membership with the protagonist. The left–right position of the two characters was counterbalanced. A gender-neutral name such as Ji Mi/几米 was given to the protagonist for each picture card.

**Figure 3:**
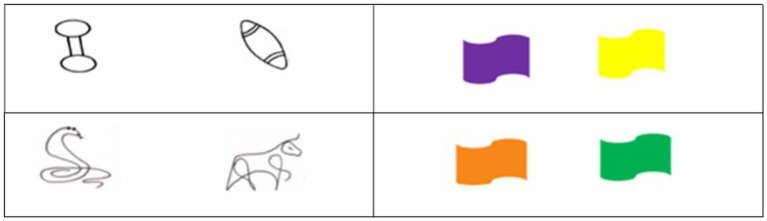
Objects of preference and flags used in Task 3.

A new set of objects and groups was used to avoid potential confusion caused by repeated use of the same sets. Representations of novel toys and pets similar to those used in previous studies (e.g., Roberts and Horii, 2019) were employed with two new pairs of color to indicate the groups (Figure 3).

**Figure 4:**
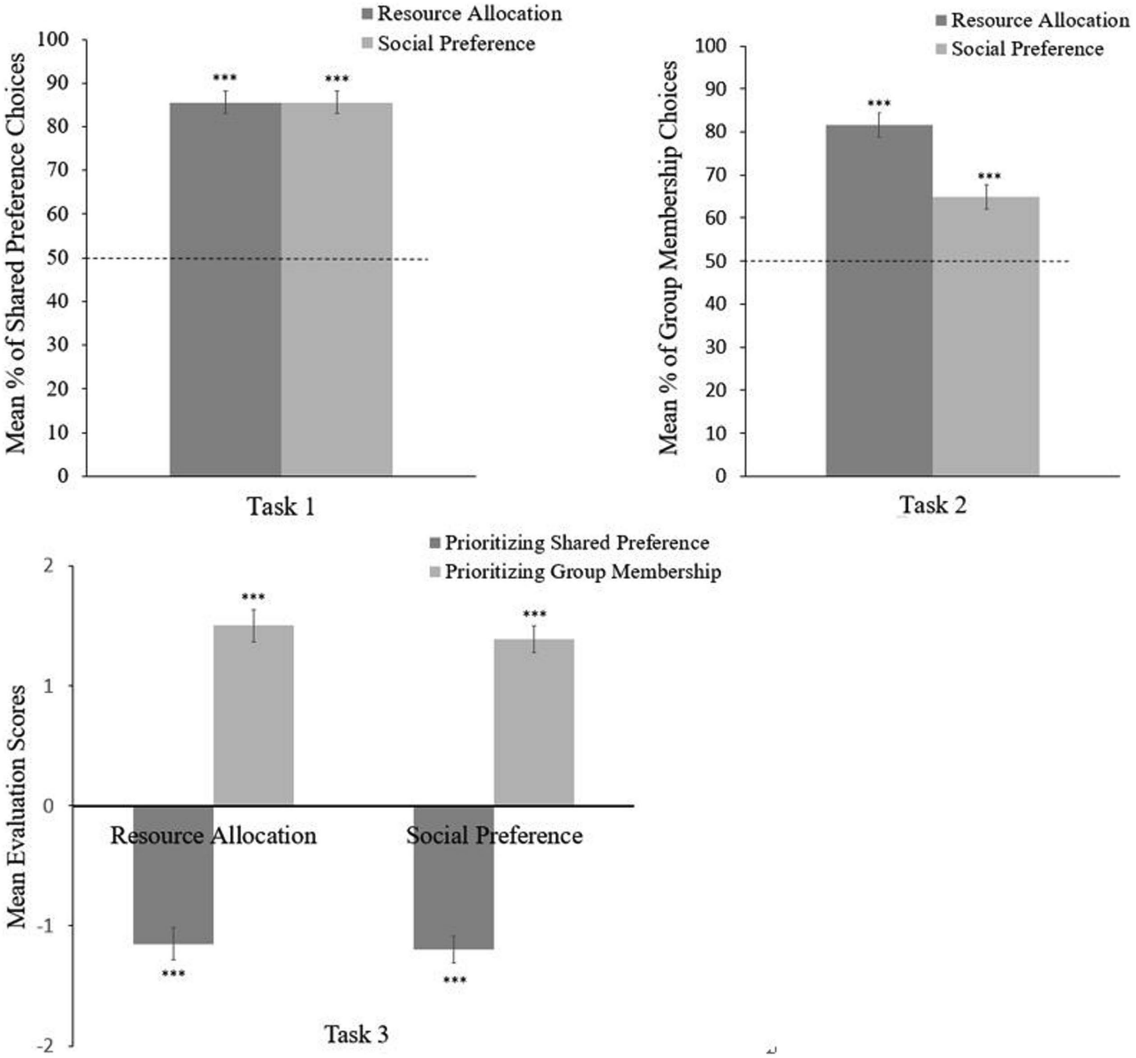
Results of Task 1 (upper left), Task 2 (upper right), and Task 3 (bottom). Error bars depict ±1 standard error, and asterisks indicate means that are significantly different from chance (^***^p < 0.001).

Four of the eight picture cards depicted how the protagonist distributed the three stickers to the other two characters, and the other four depicted the protagonist’s liking indicated by heart over their preferred character. Two of the four distribution picture cards showed resource allocation biased in favor of shared preference over group membership, while the other two showed a bias in favor of group membership over shared preference. Of the four liking cards that indicated the protagonist’s character preference, two favored the character who shared preferences, while the other two favored the character who shared group membership.

### Procedure

The procedure of this study was approved by the Bioethics Review Committee of Seoul National University (IRB No. 2012/003–016). Data collection took place in a kindergarten in Zhangjiakou City, Hebei Province, China. We first obtained written permission from the head of the kindergarten after explaining the purpose and procedure of the study. Study descriptions, consent forms, and questionnaires were described to the teachers who distributed the material to the children’s guardians. Only children who expressed interest and whose guardians provided written consent participated in this study.

Children were individually tested in a quiet room in a kindergarten. The researcher had a casual conversation with the child before the tasks to build rapport. A practice session was initiated once the child felt comfortable. In the practice session, the child was presented with three stickers and a picture card showing a panda and a frog. The child was then told to distribute the stickers between the animals freely but to use up all the stickers. The researcher ensured that all stickers were distributed. After that, the researcher asked the child which of the two animals he/she wanted to be friends with but instructed him/her to choose only one. This practice session was followed by the three tasks described below. The tasks were presented in a fixed order, at advancing levels of complexity, to facilitate children’s understanding of the tasks. The pilot test (conducted with an additional five children) suggested that some children might have difficulty understanding Task 3 (a relatively complex task) when it was presented as the first task of the experiment. For all tasks, children’s responses were recorded by the researcher on video and a test sheet.

The first task tested resource allocation and social preference based on shared preferences. The researcher presented the child with a pair of toys (or pets) and asked which of the two items he/she would like to play with more. After ascertaining the child’s preference, she showed the child a picture card depicting two characters and introduced a character who shared the child’s preference and a character who did not. The researcher indicated which character liked to play with the shared preference and which character liked to play with an item that the child did not choose, putting the laminated pictures of the items next to the characters. Then, the researcher requested the child to distribute the stickers between the characters explaining that they should use all the stickers. Once the child distributed the stickers to the characters, the researcher asked the child which character he/she wanted to be friends with. The resource allocation always preceded the friend choice question as in previous work (Renno and Shutts, 2015) so that children’s decisions in resource allocation could not be influenced by their friend choice. After the child had allocated the stickers or selected a friend, the researcher verbally confirmed the responses. Each child received four trials for this task, distributing the resources four times and choosing friends four times. Two trials presented the child with pairs of toys, and the remaining two presented them with pairs of pets as items of preference.

Task 1 was followed by Task 2. The researcher showed the child two different color flags and told him/her that whichever color he/she was given was the color class he/she would belong to. The child was then handed a flag and thereby assigned to a group (e.g., a sky blue class). The color of the flag given to the child was counterbalanced. Next, the researcher introduced two characters to the child, using a picture card. One character introduced shared a preference with the child but not the child’s group; the other character introduced shared a group with the child, but not their preference. The researcher then asked the child to confirm which character shared the child’s preference and which character shared the child’s group to demonstrate understanding. If answered incorrectly, the child was reinformed of the characters’ status until the correct answers were given. After that, the child’s resource allocation and social liking were measured in the same way as in Task 1, by asking them to distribute three stickers between the characters and choose which they wanted to be friends with. Once the child allocated resources or selected a friend, a confirmation was made. Again, there were four trials for this task. The presentation order of the characters (i.e., Same-preference character and Same-group character) was counterbalanced within and across participants.

Once the child completed Task 2, Task 3 was carried out to test children’s evaluation of resource allocation and social preference of others. The task consisted of four trials whereby children were first asked to evaluate others’ resource allocation and then to evaluate others’ social preference. The researcher introduced the protagonist (e.g., Ji Mi) on a picture card and indicated the protagonist’s preference and group for each trial. The researcher then introduced two additional characters—a character who shared the same preference as the protagonist but belonged to a different group and a character who did not share a preference with the protagonist but belonged to the same group. Children’s understanding of the three characters, their group, and their preferences was confirmed. If the child confirmed incorrectly, the information was reinformed to ensure understanding. After confirmation of understanding, the researcher described how the protagonist distributed the stickers to the other characters. The picture card also indicated which character received more stickers than the other. She then asked the child to evaluate the protagonist’s resource allocation by asking if the behavior was good or bad and to what degree. If the child answered that the behavior was good, the researcher asked them to clarify whether it was a little good or very good. The same was asked if they answered that the behavior was bad. They were also asked to justify the evaluation. The protagonist’s preference was then evaluated. The researcher described the protagonists’ friend choice between the two characters. The child’s evaluation of the protagonist’s friend choice was measured using the same questions as those used to evaluate the protagonist’s resource allocation. After the third task, the child was thanked and given a small gift. The exact wording for the procedure of Tasks 1 to 3 is presented in the Supplementary Material.

### Scoring

For each trial of Task 1, children received one point for a response in favor of the character with a shared preference. That is, if they distributed more stickers to the character with a shared preference, or if they chose them as the one they wanted to be friends with in each trial, they received one point. Otherwise, they received no points. Thus, the possible resource allocation scores ranged from 0 to 4. The possible social preference scores ranged the same. Higher scores indicated more resource allocation and social preference in favor of the shared-preference character over the different-preference character.

For Task 2, children were given one point for responses in favor of a same-group-different-preference character over the different-group-same-preference character. That is, they received one point if they distributed more stickers to the same-group character or chose the same-group character as friends. Otherwise, they received no points. The possible scores for resource allocation and social preference based on group membership ranged from 0 to 4. Higher scores indicated more responses in favor of the same-group character over the same-preference character.

For Task 3, evaluation of the protagonist’s behavior was scored as follows: very bad was scored as −2, a little bad −1, a little good 1, and very good 2. If no clear evaluation was given, no points were assigned. Then, for each child, the average scores for the two trials in which children evaluated protagonist’s resource distribution or social preference in favor of the same-group character were obtained. Likewise, average scores for the remaining two trials in which children evaluated protagonist’s resource distribution or social preference in favor of shared preference were obtained. The averages obtained allowed for a more intuitive understanding of children’s evaluations, which could be mapped between very bad (−2) and very good (2).

## Results

### Resource Allocation and Social Preference Based on Shared Preference

Figure 4 indicates the main results of this study. Children allocated more resources to characters who shared preferences with them than to those who had different preferences in 3.42 trials (*SD* = 0.83) out of four trials, which differed significantly from chance probability, *t*(63) = 13.67, *p* < 0.001, *d* = 1.71. In addition, children chose characters who shared preferences as friends in an average of 3.42 trials (*SD* = 1.04) out of four trials. The score differed significantly from chance probability, *t*(63) = 10.98, *p* < 0.001, *d* = 1.37. Thus, children favored characters with the same preferences as them over those with different preferences in both resource allocation and social preference.

### Resource Allocation and Social Preference Based on Shared Preference vs. Group Membership

Children distributed more resources to characters in the same group who did not share preferences than to characters in a different group who shared preferences, in an average of 3.27 (*SD* = 0.91) out of four trials. The score differed significantly from chance, *t*(63) = 11.09, *p* < 0.001, *d* = 1.40. Also, children chose to be friends with characters from the same group who did not share their preferences in an average of 2.59 (*SD* = 1.35) trials, which differed significantly from the chance probability, *t*(63) = 3.51, *p* < 0.001, *d* = 0.44. Therefore, children prioritized group membership over shared preferences in their distribution of resources and friend choice.

### Third-Party Evaluation on Others’ Resource Allocation and Social Preference

When shared preference and group membership conflicted with one another, the average evaluation score for resource allocation that prioritized shared preferences was −1.15 (*SD* = 1.05). The score differed significantly from zero, *t*(63) = 8.76, *p* < 0.001, *d* = −1.10, indicating that children evaluated the behavior as negative. In contrast, the average evaluation score for resource allocation prioritizing group membership was 1.50 (*SD* = 0.89), which differed significantly from zero, *t*(63) = 13.54, *p* < 0.001, *d* = 1.69, indicating a positive evaluation of the behavior. These scores differed significantly, demonstrating that children evaluated resource allocation that prioritized group membership more positively than resource allocation prioritizing shared preference from a third-party standpoint, *t*(63) = 16.12, *p* < 0.001, Hedges’ *g* = 2.69.

The mean evaluation score for others’ social preference that prioritized shared preference was −1.20 (*SD* = 1.14), which was significantly different from zero, *t*(63) = 8.39, *p* < 0.001, *d* = −1.05. Thus, children evaluated the preference as negative. However, the average evaluation score for preference prioritizing group membership was 1.39 (*SD* = 1.04), significantly greater than zero, *t*(63) = 10.69, *p* < 0.001, *d* = 1.34, indicating positive evaluation of the preference. Children favored prioritization of group membership over prioritizing shared preference when shared preference and group membership were in conflict as a third party, *t*(63) = 14.04, *p* < 0.001, Hedges’ *g* = 2.35.

## Discussion

This study was designed to test (1) whether five-year-old children would consider shared preference for their resource allocation and social liking, (2) whether five-year-old children would prioritize shared preference or group membership for resource allocation and social liking when the two are in conflict, and (3) whether the priority would generalize to children’s third-party evaluation of the resource allocation and liking of others.

First, five-year-old children allocated more resources to and showed more liking toward the characters who shared their preferences than those who had different preferences. These results indicate that children take shared preference into account for both resource allocation and social liking at age five, consistent with earlier findings with younger children’s (e.g., Fawcett and Markson, 2010b), same-age peers’ (Rekalidou and Petrogiannis, 2012; Sparks et al., 2017), older children’s (Heiphetz et al., 2014), and adults’ (Vélez et al., 2019) social liking. The results are also in line with young children’s resource sharing in Sparks et al. (2017). In addition, it extends previous findings showing the effect of shared preferences on young children’s affiliation and generosity, from children in Western countries to children in an East Asian country. Moreover, this finding, combined with previous findings showing that the recipient’s group membership affects young children’s resource allocation and social liking (e.g., Dunham et al., 2011; Sparks et al., 2017; Yang and Dunham, 2019), suggests that shared preference and group membership can be placed in competition as factors of resource allocation and social preference in young children.

However, the present study cannot tell us about the mechanisms underlying the affiliation with and generosity to similar-preference individuals. The mechanisms may relate to an emotional sense of connectedness that interpersonal similarity might bring about Dishion et al. (1994). There may be more strategic motives as well. For instance, children may care about shared preference because it is a positive sign for a possible friendship (Fawcett and Markson, 2010b) and investing in a potential friendship is more promising (Sparks et al., 2017). Exploration of children’s reasoning about shared preferences would be an interesting avenue for future research.

Our second finding concerns children’s relative weighing in forced-choice scenarios of resource allocation and social liking when shared preference and group membership are in conflict. Five-year-old children distributed more resources to and showed more liking toward characters in the same group who did not share their preferences than those from a different group who shared their preferences. This finding is consistent with the earlier finding by Sudo (2021) in that children do not favor the out-group over their in-group despite the shared preference. However, going beyond that, our study provides the first evidence that young children actually favor the in-group with dissimilar tastes over the out-group with similar tastes, when required to choose one person to befriend or benefit more in resource allocation. Thus, when the equality rule cannot be followed, children privilege group membership over shared preference in both resource allocation and social liking.

The current finding corroborates five-year-old IGF in resource allocation (Dunham et al., 2011; Plötner et al., 2015a; Sparks et al., 2017) and social preference (Dunham et al., 2011; Sparks et al., 2017; Yang and Dunham, 2019), adding new information about its importance relative to shared preference. In addition, it is comparable to the prioritization of group membership over shared preferences in older children (Nesdale et al., 2010). Thus, our finding suggests that attaching greater importance to belonging to the same group than having some preferences in common in selective favorable behaviors is already evident by the age of five.

Another novel finding of this study is that five-year-old children’s prioritization of group membership over shared preference translates to their third-party evaluation of others. Five-year-old children positively regarded resource allocation and social liking in others that prioritized group membership over a shared preference, whereas they evaluated behaviors that prioritized shared preference over group membership negatively. Importantly, children were affiliated with neither group in the task and were not influenced by the protagonist’s resource allocation and friend choice. Thus, children’s evaluation in the present study is likely to apply to others in general (i.e., in an agent-neutral way). The result then provides initial evidence suggesting that five-year-old children possess a normative stance that the priority to group membership over shared preference is something good and more appropriate than the reverse priority if the two are in conflict.

Our finding that children regard others’ prioritization of group membership more positively than prioritization of shared interest from the perspective of a third person is compatible with the prior findings that group loyalty is an expected norm for five-year-old children (Misch et al., 2014, 2016, 2018; Jin et al., 2019). Perhaps, becoming a reliable member of a social group may be a more important issue for five-year-old children, relative to affiliating with and being generous to individuals with similar preferences, as suggested by the social identity development theory (Nesdale, 2004).

In the present study, we have provided the analysis of children’s justifications of social evaluations in Supplementary Material. A large proportion of the responses refer to the group membership and preference information given by the researcher (e.g., “Because this child is in the same class,” “They are not classmates,” and “They like different pets”). However, some responses offered interesting explanations for their endorsement of group membership prioritization. As justification for distributing more resources to in-group members, children indicated both an expectation of reciprocity among in-group members and a belief that benefitting in-group members is a normative behavior. For example, children said that by distributing more resources to in-group members, they could receive help from them. They also indicated that giving more resources to in-group members was “a kind of duty,” and “normal,” “natural,” or the “right” behavior. On the contrary, allocating more resources to out-group members was seen as the “wrong behavior.” These justifications are consistent with the previous finding that five- to thirteen-year-olds judged that characters would feel more obligated to help an unfamiliar child from an in-group than an out-group (Weller and Lagattuta, 2013). In addition, some children referred to out-group members as an “out person (外人)” and said that giving them only one sticker should be okay. In contrast, they called an in-group member a “companion (同伴)” and said that giving the companion only one sticker should not be okay. As justifications for playing with in-group members, children mentioned that playing with classmates was the most “appropriate” and “correct” behavior. One child mentioned that other classmates might disapprove of the protagonist if the protagonist did not choose the same-class member as a friend. Lastly, a few children deemed that being in the same class was a prerequisite for being friends. Thus, in fact, children’s normative stance about the priority to group membership is also found in their justifications of social evaluations.

Altogether, our findings indicate that group membership weighs more than shared preference in young children’s selective actions (resource allocation and liking) and their third-party appraisal of the actions of others. However, several limitations should be noted about the current study. It only tested five-year-old children and could not speak to possible developmental changes. Comparing the findings from multiple studies indicates that five- to eight-year-olds, but not three- to four-year-olds, show in-group preference in minimally defined groups (Dunham et al., 2011; Dunham and Emory, 2014; Yang and Dunham, 2019). Furthermore, a positive evaluation bias for in-group members is evident in six-year-olds but not in three-year-olds (Dunham and Emory, 2014). Future research should examine developmental changes in children’s tendency to prioritize group membership over shared preference. Second, our findings may not apply to children from other cultures as this study was conducted only on Chinese children living in China. Prioritization of group membership may differ in children from an individualistic culture that emphasizes self-direction based on individuals’ desires, preferences, and needs. Those in a collectivist culture may emphasize the maintenance of group harmony based on in-group cohesion and the duties imposed by the collective (Schreier et al., 2010; Yu et al., 2016; Over and McCall, 2018; Triandis, 2018). Further studies should be conducted on children from diverse cultures such as Korea and the United States. Third, our study presented characters as drawings rather than photos or actual children. While drawings can control for potential variables of characters that may influence children’s responses, the results might not be representative of their responses to actual peers. Future studies should investigate children’s resource allocation, social preference, and evaluation in a more naturalistic setting. Additionally, while children were distributing the resources and choosing friends, the researcher ensured their safety and adherence to the distribution and friend choice rules. Although the researcher responded to the children’s behaviors neutrally, the researcher’s presence might have influenced the children’s responses in the tasks. Nonetheless, we consider it unlikely that the findings would change in the absence of the researcher, considering that most children in our study responded to the task without any hesitation. Also, although not all children were able to articulate their reasons underlying the evaluations, there were responses that clearly justify the prioritization of group membership over shared preference. Last but not least, it is important to note that the current findings were obtained from forced-choice paradigms. The forced-choice format represents only a part of real life situations. Children in real life may be given more diverse options; for instance, they may distribute resources to others and keep the remaining sticker with them. Thus, generalization of the current findings is limited. Nevertheless, it is true that children sometimes encounter such situations in which they have to selectively benefit or approach others in their lives. Our study aimed to focus on the relative importance of shared preference versus group membership and suggests that children may regard group membership over shared preferences in such contexts.

Despite the limitations, the present study fills the gap in prior work on the impact of shared preference and group membership on children’s social preference and resource allocation, by presenting a situation in which shared preferences are in conflict with group membership and children have to favor one over the other in their liking and resource allocation. Also, this study is the first to examine five-year-old children’s evaluation of resource allocation and preference by unaffiliated others, where either shared preference or group membership were prioritized in a third-party context. Lastly, most previous work on children’s group-mindedness was conducted in individualistic western countries. This study fills the gap by testing children living in an eastern collectivist country and provides a stepping stone for cross-cultural research to better understand the development of group-mindedness in children from different cultural backgrounds.

As a whole, the study elucidates children’s developing group-mindedness. First, children readily recognized the group division even though the groups were previously unfamiliar ones, as evident in not only their correct answers to the confirmation questions (e.g., “I am in the sky blue class.”) but also their frequent, spontaneous use of distinguishing labels during the task (e.g., “I have to give a lot to *my class*” and “I should give *my* class two, and *their* class one.”). Second, they demonstrated more favorable social behaviors to their in-group than out-group members despite the shared preferences with the out-group members. Third, their justification for evaluating others’ resource allocation and preference reveals that children pay attention to and abide by group norms, as indicated by their remarks that a favorable behavior toward in-group members is normal and that a more favorable behavior toward out-group members is considered wrong and carries the cost of social rejection.

In conclusion, although shared preference affects children’s resource allocation and social preference at the age of five, young children attach greater importance to group membership than shared preference in their selective resource allocation and social liking when the two are in conflict. Further, this priority translates to their evaluation of resource allocation and social preference of others as a third party. Our findings, together with other converging evidence from five-year-olds (Dunham et al., 2011; Misch et al., 2014, 2016, 2018; Plötner et al., 2015a; Sparks et al., 2017; Jin et al., 2019; Yang and Dunham, 2019), suggest that, by five years of age, children are already developing a strong sense of group-mindedness, with group membership playing a crucial role in their social behavior and peer evaluation.

## Data Availability Statement

The raw data supporting the conclusions of this article will be made available by the authors, without undue reservation.

## Ethics Statement

The studies involving human participants were reviewed and approved by the Bioethics Review Committee of Seoul National University (IRB No. 2012/003–016). Written informed consent to participate in this study was provided by the participants’ legal guardian/next of kin.

## Author Contributions

LY collected and organized the data. All authors contributed to conception and design of the study, performed the statistical analysis, and wrote the manuscript. All authors contributed to the article and approved the submitted version.

## Conflict of Interest

The authors declare that the research was conducted in the absence of any commercial or financial relationships that could be construed as a potential conflict of interest.

## Publisher’s Note

All claims expressed in this article are solely those of the authors and do not necessarily represent those of their affiliated organizations, or those of the publisher, the editors and the reviewers. Any product that may be evaluated in this article, or claim that may be made by its manufacturer, is not guaranteed or endorsed by the publisher.
