# Supplementary material for: Group Membership Trumps Shared Preference in Five-Year-Olds’ Resource Allocation, Social Preference, and Social Evaluation
Source: Front Psychol. 2022 May 31;13:866966. doi: 10.3389/fpsyg.2022.866966 (PMC9197506; doi:10.3389/fpsyg.2022.866966)
Supplement: Supplementary file 1 [file Presentation_1.pdf]

Group Membership Trumps Shared Preference in Five-Year-Olds' Resource Allocation, Social Preference, and Social Evaluation

Supplementary Material

Li Yang[a] and Youjeong Park[a]

Department of Child Development and Family Studies, Seoul National University, Seoul, South Korea

Correspondence: Youjeong Park

youjeongpark@snu.ac.kr

S1. Script for the procedure

S2. Number of stickers distributed in Tasks 1 and 2

S3. Categories and frequencies (in Percent) of children's justifications in Task 3

S1. Script for the Procedure

A practice trial

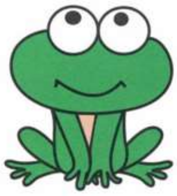

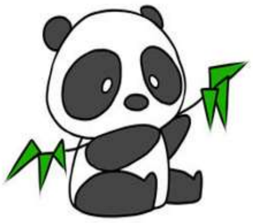

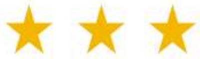

Here are two animals. You can give these stickers to the animals now. You should use up all of the stickers.

Do you want to try giving these stickers to the two animals?

(Child allocates stickers.)

Good! You gave this frog X and this panda Y. You don’t have any stickers left, right?

(If the child does not distribute all of the stickers, the experimenter explains the rule again until he/she fully understands the rule.)

Now, which of these animals do you want to be friends with? Please tell me only one animal.

(Child chooses a friend.)

Good! You chose the panda/frog. You chose only one animal, right?

(If the child does not choose only one animal, the experimenter explains the rule again until he/she fully understands the rule.)

Task 1

1) A pair of toys (or pets)

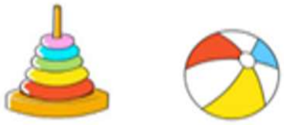

Here are two toys. Which toy do you like to play with more?

2) A pair of toys (or pets) and two characters

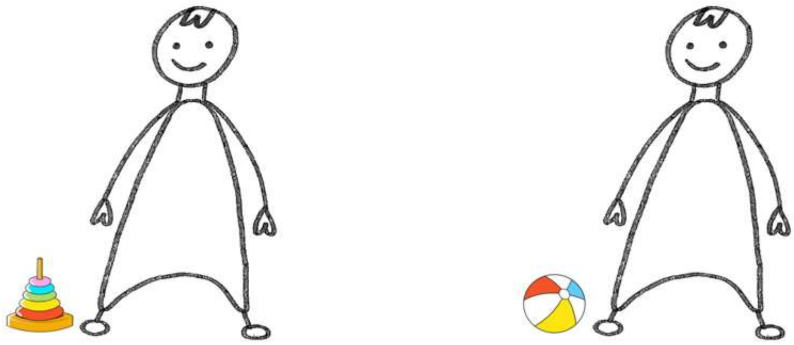

This kid (pointing to a character) likes to play with □□ (the toy that the child chose). And this kid (pointing to the other character) likes to play with △△ (the toy that the child did not choose).

3) Stickers

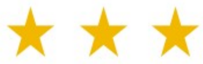

Can you give these stickers to the two kids? You should use up all the stickers.

(Child allocates the stickers.)

You gave this kid X and this kid Y. Do you want to give them like this?

Which one do you want to be friends with?

(Child chooses a friend)

Do you want to make friends with this kid?

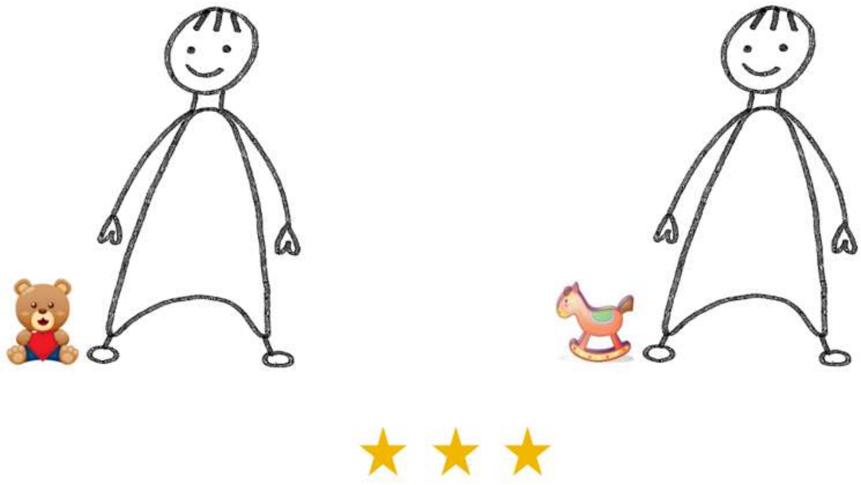

Here are two toys too. Which toy do you like to play with more?

This kid (pointing to a character) likes to play with □□ (the toy that the child did not choose). And this kid (pointing to the other character) likes to play with △△ (the toy that the child chose).

Can you give these stickers to the two kids? You should use up all the stickers.

(Child allocates stickers)

You gave this kid X and this kid Y. Do you want to give them like this?

Which one do you want to be friends with?

(Child chooses a friend)

Do you want to make friends with this kid?

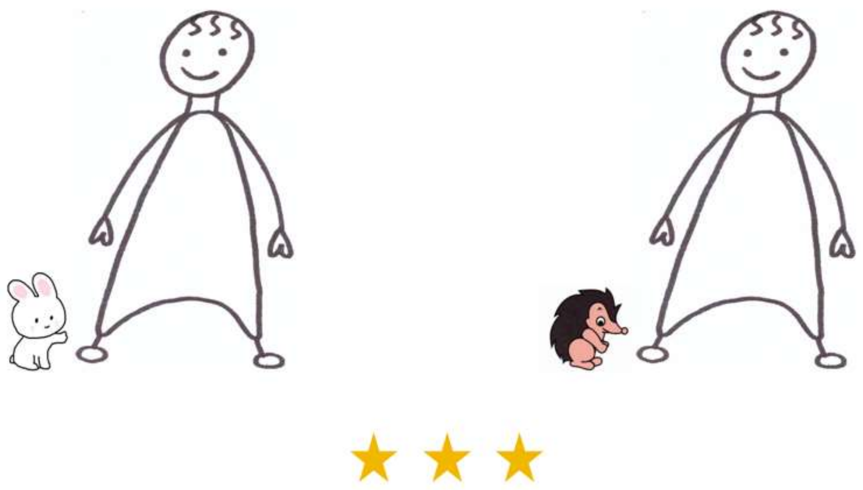

Here are two pets. Which kind of pet do you like to keep? This kid (pointing to a character) likes to keep □□ (the pet that the child chose). And this kid (pointing to the other character) likes to keep △△ (the pet that the child did not choose).

Can you give these stickers to the two kids? You should use up all the stickers.

(Child allocates stickers)

You gave this kid X and this kid Y. Do you want to give them like this?

Which one do you want to be friends with?

(Child chooses a friend)

Do you want to make friends with this kid?

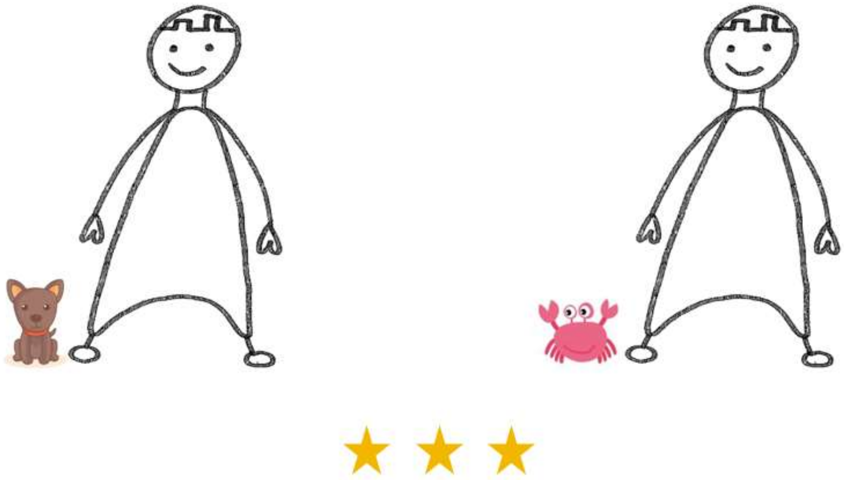

Here are two pets too. Which kind of pet do you like to keep? This kid (pointing to a character) likes to keep □□ (the pet that the child did not choose). And this kid (pointing to the other character) likes to keep △△ (the pet that the child chose).

Can you give these stickers to the two kids? You should use up all the stickers.

(Child allocates stickers)

You gave this kid X and this kid Y. Do you want to give them like this?

Which one do you want to be friends with?

(Child chooses a friend)

Do you want to make friends with this kid?

Task 2

1) A pair of color flags

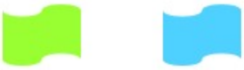

Now here are two colors of flags. One is a light green flag and the other is a sky blue flag (showing the child the actual flags). I will give you one of these flags. If you receive a light green flag, you will belong to the Light Green class, and if you receive a sky blue flag, you will belong to the Sky Blue class. Okay?

2) Two characters

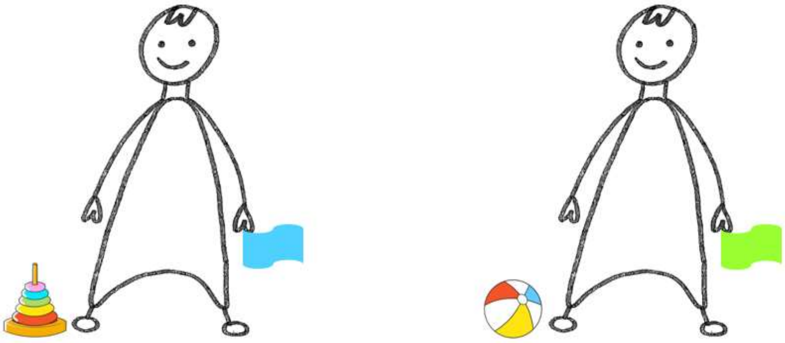

This kid likes to play with □□ (the toy that the child chose). And he is in the Sky Blue class (the class that the child does not belong to). This kid likes to play with △△ (the toy that child did not choose). And he is in the Light Green class (the child's class). Which one likes to play with the same toy as you? Who is in the same class with you?

3) Stickers

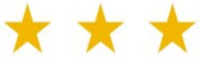

Can you give these stickers to the two kids? You should use up all the stickers. (Child allocates stickers) You gave this kid X and this kid Y. Do you want to give them like this?

Which one do you want to be friends with? (Child chooses a friend) Do you want to make friends with this kid?

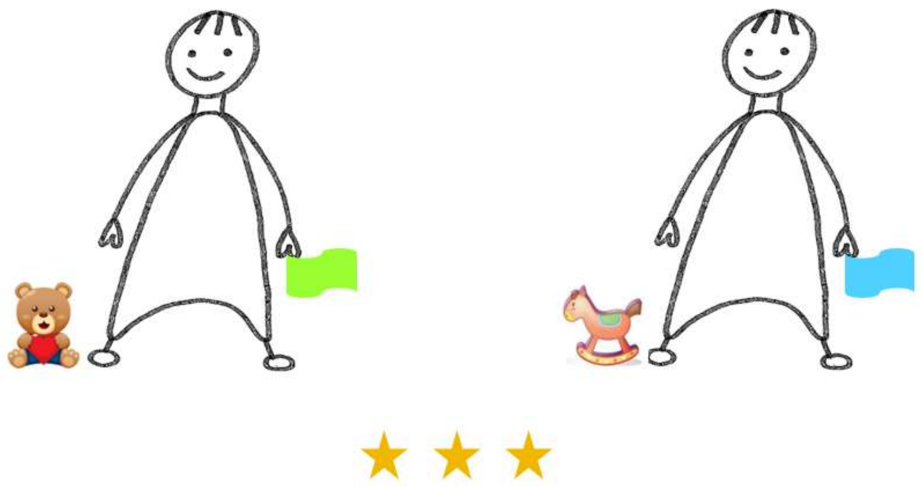

This kid likes to play with □□ (the toy that the child did not choose). And he is in the Light Green class (the child’s class). This kid likes to play with △△ (the toy that child chose). And he is in the Sky Blue class (the class that the child does not belong to).

Which one likes to play with the same toy as you?

Who is in the same class with you?

Can you give the stickers to the two kids?

(Child allocates stickers)

You gave this kid X and this kid Y. Do you want to give them like this?

Which one do you want to be friends with?

(Child chooses a friend)

Do you want to make friends with this kid?

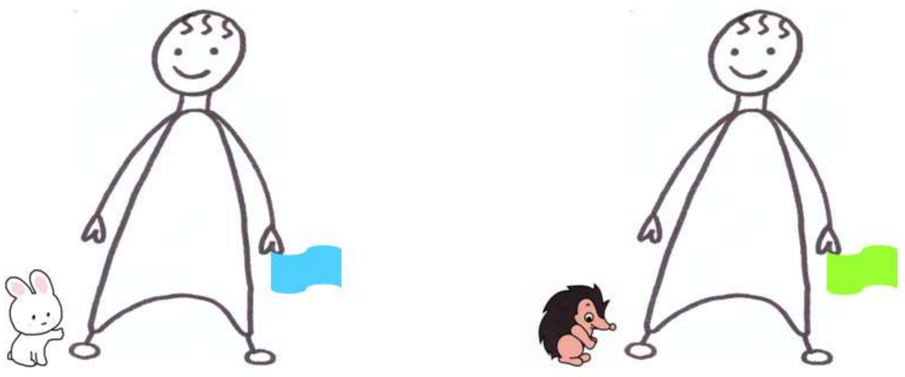

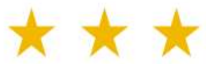

This kid likes to keep □□ (the pet that the child chose). And he is in the Sky Blue class (the class that the child does not belong to). This kid likes to keep △△ (the pet that child did not choose). And he is in the Light Green class (the child's class).

Which one likes to keep the same pet as you?

Who is in the same class with you?

Can you give the stickers to the two kids?

(Child allocates stickers)

You gave this kid X and this kid Y. Do you want to give them like this?

Which one do you want to be friends with?

(Child chooses a friend)

Do you want to make friends with this kid?

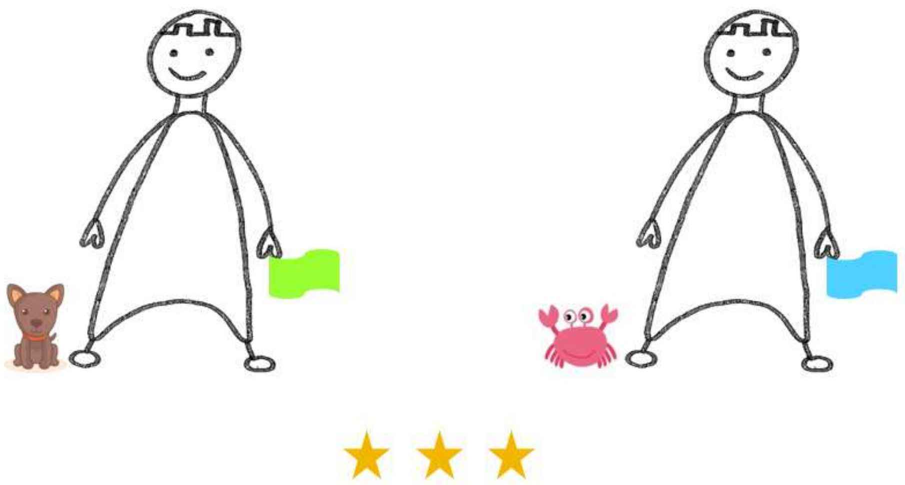

This kid likes to keep □□ (the pet that the child did not choose). And he is in the Light Green class (the child's class). This kid likes to keep △△ (the pet that child chose). And he is in the Sky Blue class (the class that the child does not belong to).

Which one likes to keep the same pet as you?

Who is in the same class with you?

Can you give the stickers to the two kids?

(Child allocates stickers)

You gave this kid X and this kid Y. Do you want to give them like this?

Which one do you want to be friends with?

(Child chooses a friend)

Do you want to make friends with this kid?

Task 3

1) Three characters with toys, flags, and stickers

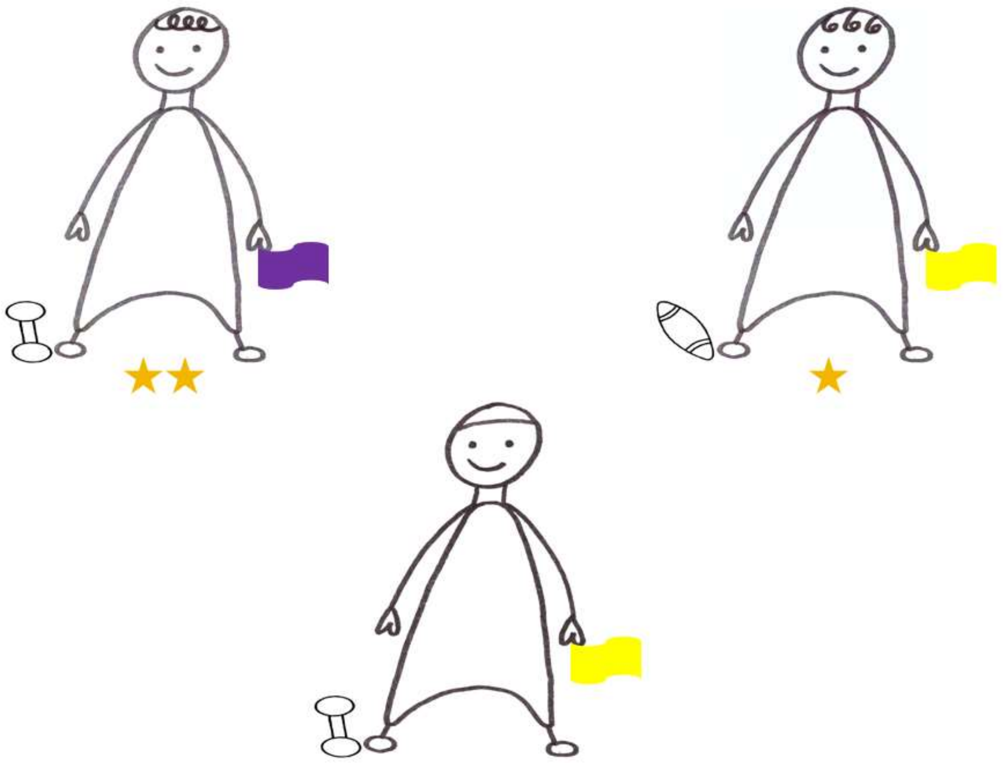

This kid (pointing to the character at the bottom) is Ji Mi. He/she likes to play with this kind of toy, and he/she is in the Yellow class.

This kid (the upper left character) likes to play with this kind of toy, and he/she is in the Purple class.

And this kid (the upper right character) likes to play with this kind of toy, and he/she is in the Yellow class.

Who likes to play with the same toy as Ji Mi?

Who is in the same class with Ji Mi?

Now, Ji Mi has given out stickers to the two kids. He/she gave this kid two, and this kid one.

Is Ji Mi's behavior good or bad?

(If the child answers "Good") Is it a little good or very good?

(If the child answers "Bad") Is it a little bad or very bad?

Why do you think so?

2) Three characters with toys, flags, and a heart shape (friend choice)

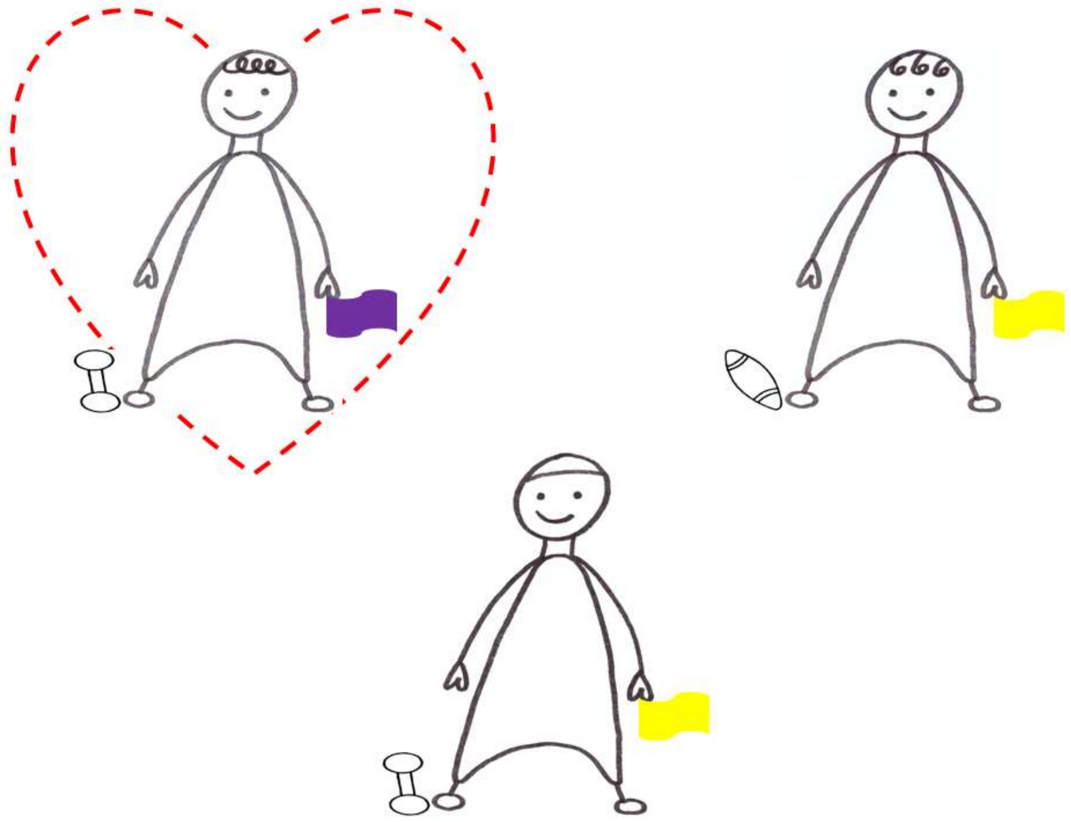

Ji Mi said he/she wanted to be friends with this kid (a heart shape) more than that kid.

Is Ji Mi's behavior good or bad?

(If the child answers "Good") Is it a little good or very good?

(If the child answers "Bad") Is it a little bad or very bad?

Why do you think so?

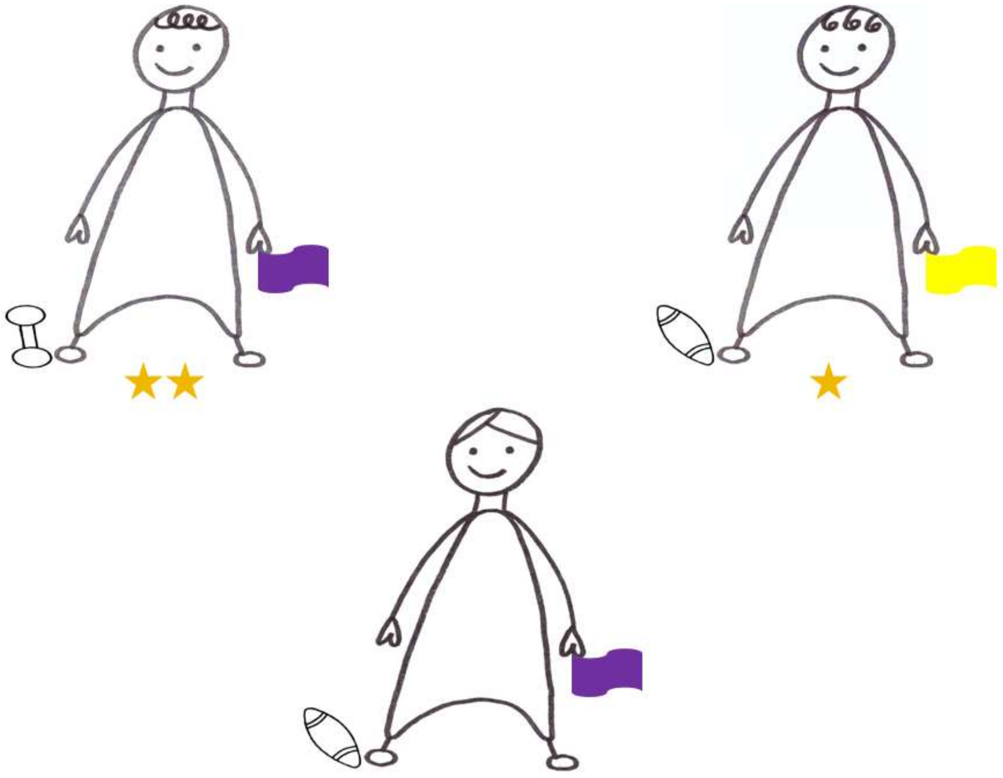

This kid is Jing Jing. He/she likes to play with this kind of toy, and he/she is in the Purple class.

This kid likes to play with this kind of toy, and he/she is in the Purple class.

And this kid likes to play with this kind of toy, and he/she is in the Yellow class.

Who likes to play with the same toy as Jing Jing?

Who is in the same class with Jing Jing?

Now, Jing Jing has given out stickers to the two kids. He/she gave this kid two, and this kid one.

Is Jing Jing's behavior good or bad?

(If the child answers "Good") Is it a little good or very good?

(If the child answers "Bad") Is it a little bad or very bad?

Why do you think so?

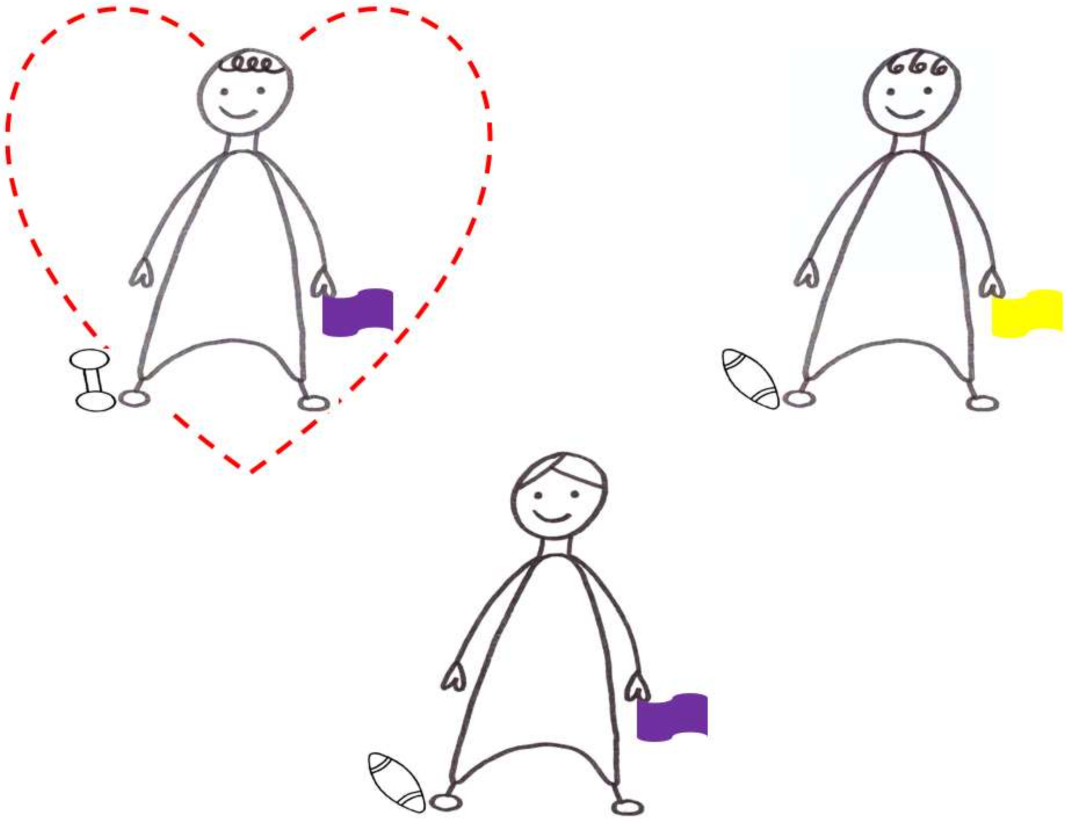

Jing Jing said he/she wanted to be friends with this kid more than that kid.

Is Jing Jing's behavior good or bad?

(If the child answers "Good") Is it a little good or very good?

(If the child answers "Bad") Is it a little bad or very bad?

Why do you think so?

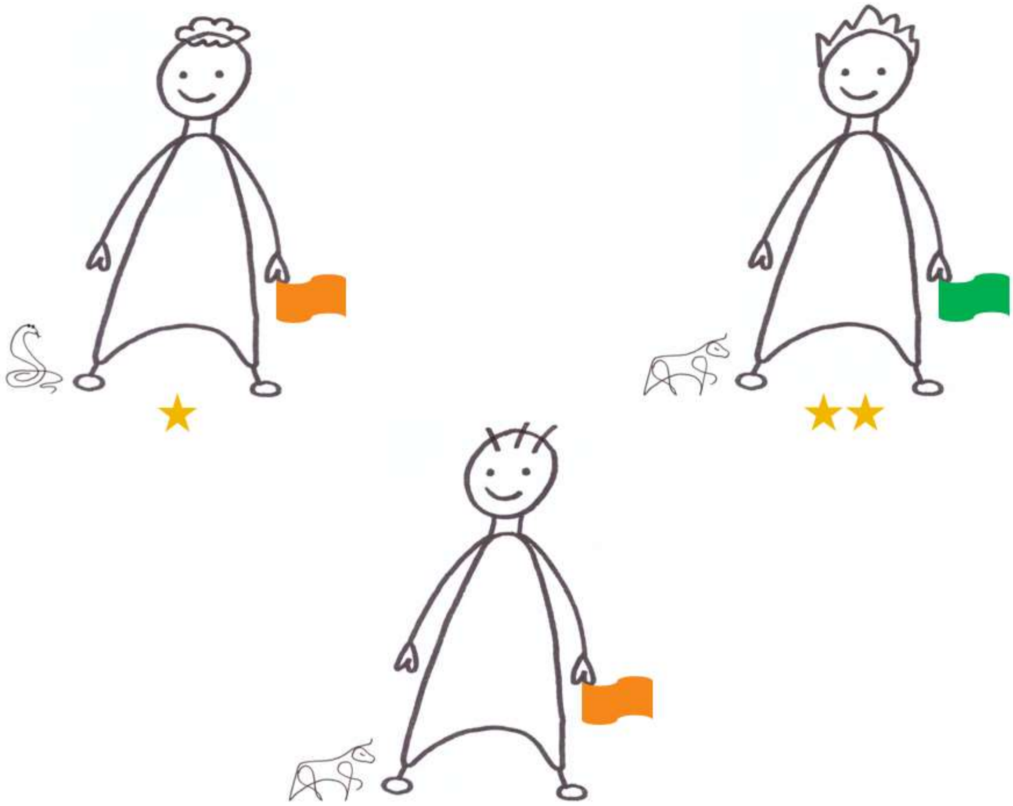

This kid is Qi Qi. He/she likes to keep this kind of pet, and he/she is in the Orange class.

This kid likes to keep this kind of pet, and he/she is in the Orange class.

And this kid likes to keep this kind of pet, and he/she is in the Green class.

Who likes to keep the same pet as Qi Qi?

Who is in the same class with Qi Qi?

Now, Qi Qi has given out stickers to the two kids. He/she gave this kid one, and this kid two.

Is Qi Qi's behavior good or bad?

(If the child answers "Good") Is it a little good or very good?

(If the child answers "Bad") Is it a little bad or very bad?

Why do you think so?

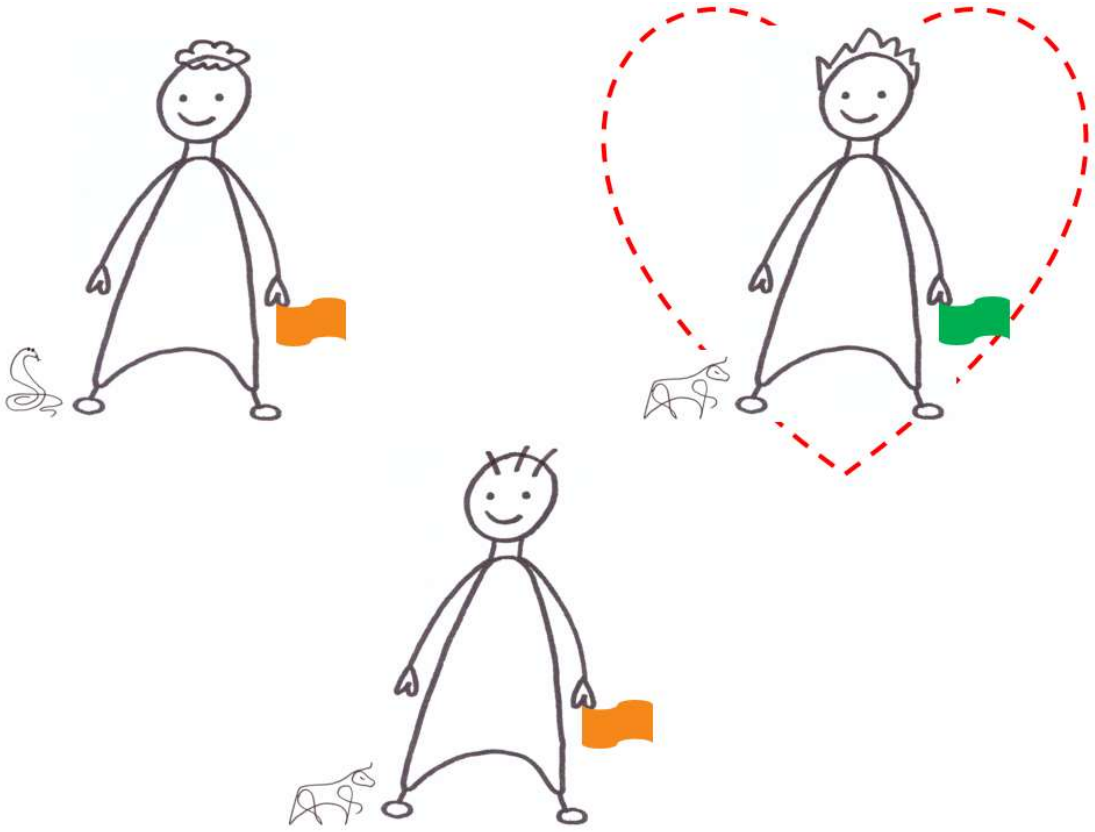

Qi Qi said he/she wanted to be friends with this kid more than that kid.

Is Qi Qi's behavior good or bad?

(If the child answers "Good") Is it a little good or very good?

(If the child answers "Bad") Is it a little bad or very bad?

Why do you think so?

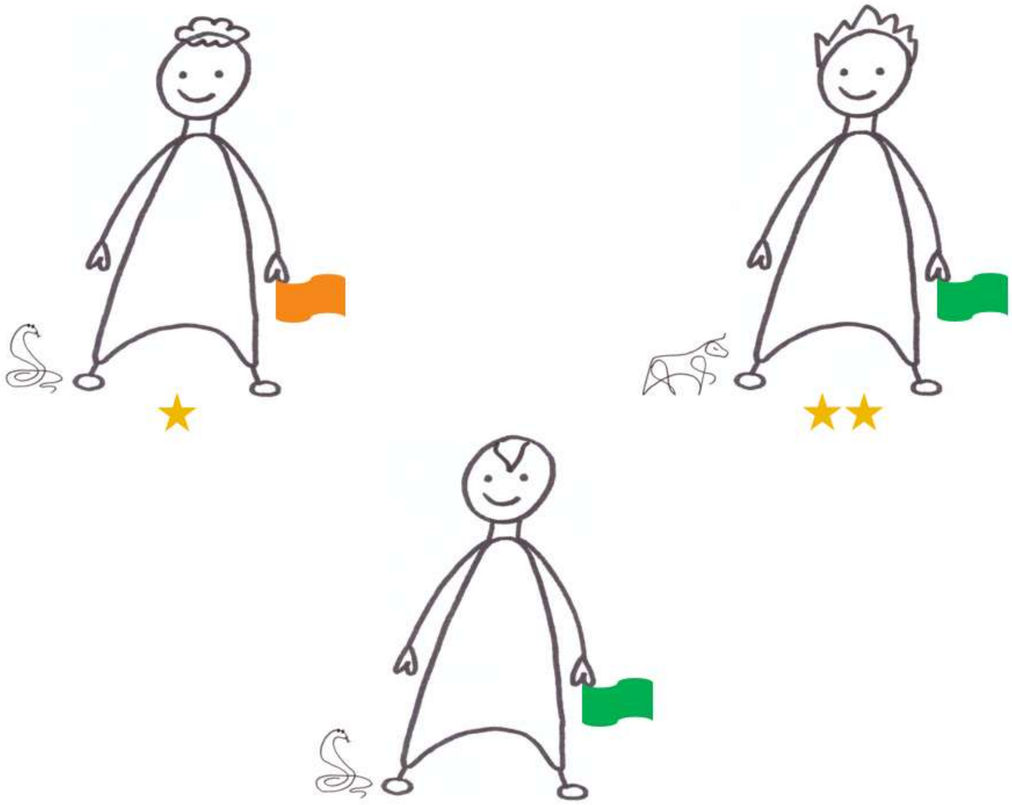

This kid is Jian Jian. He/she likes to keep this kind of pet, and he/she is in the green class.

This kid likes to keep this kind of pet, and he/she is in the Orange class.

And this kid likes to keep this kind of pet, and he/she is in the Green class.

Who likes to keep the same pet as Jian Jian?

Who is in the same class with Jian Jian?

Now, Jian Jian has given out stickers to the two kids. He/she gave this kid one, and this kid two.

Is Jian Jian's behavior good or bad?

(If the child answers "Good") Is it a little good or very good?

(If the child answers "Bad") Is it a little bad or very bad?

Why do you think so?

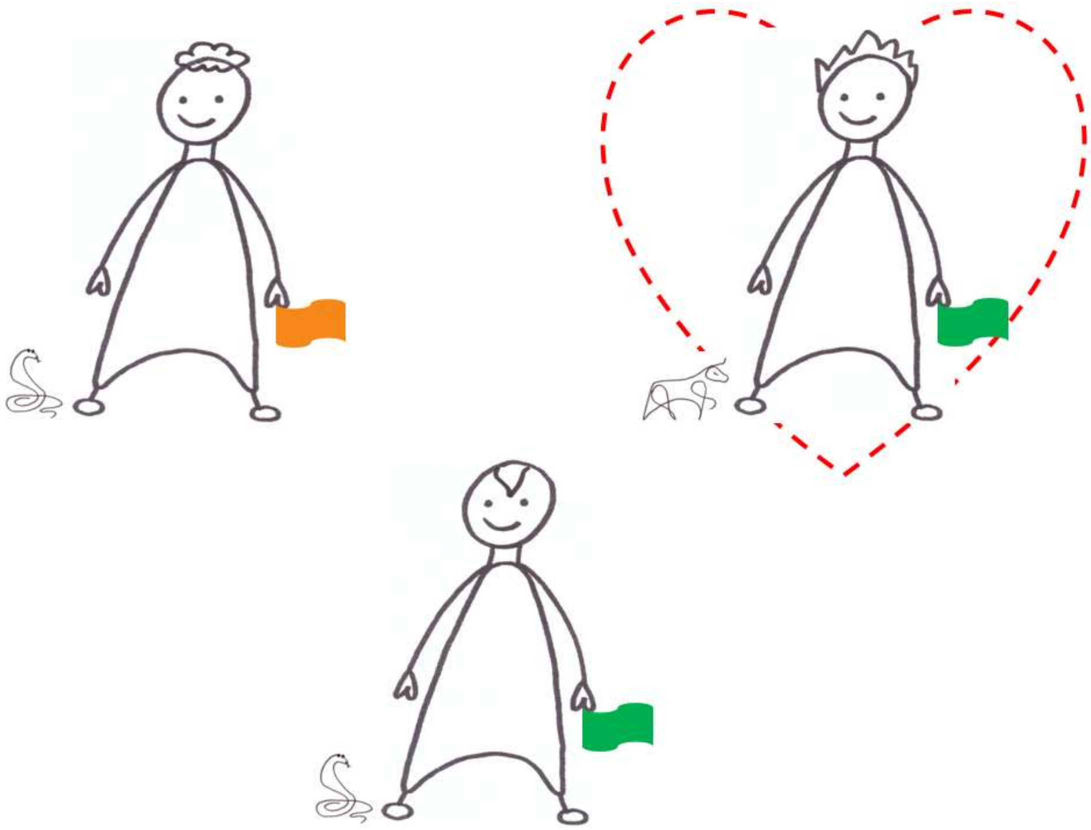

Jian Jian said he/she wanted to be friends with this kid more than that kid.

Is Jian Jian's behavior good or bad?

(If the child answers "Good") Is it a little good or very good?

(If the child answers "Bad") Is it a little bad or very bad?

Why do you think so?

S2. Number of Stickers Distributed in Tasks 1 and 2

As can be seen in Table 1, children in Task 1 distributed an average of 1.94 stickers to the characters who shared their preferences (*SD* = 0.34), which was significantly above the chance, $t(63) = 10.26$, $p < .001$. In Task 2, they distributed 1.89 stickers to the in-group characters with different preferences (*SD* = 0.36), significantly above the chance, $t(63) = 8.78$, $p < .001$.

Table 1. Average number of stickers distributed in Tasks 1 and 2

	Recipient	*M*	*SD*	Min.	Max.
Task 1	Same-preference characters	1.94	0.34	1	3
Task 2	Same-group-different-preference characters	1.89	0.36	1	3

In every trial of Task 1, all children gave at least one sticker to the characters who shared their preferences, with most children distributing two stickers to the same-preference characters (see Table 2). In each trial, approximately 7.8~9.4% of the children gave all of the three stickers to the same-preference individuals.

In Task 2, where the same-preference characters turned out to be an out-group member, most children switched their favorable distribution to the in-group members who did not share their preferences. In every trial, a vast majority of the children distributed only one sticker to the same-preference characters, distributing two stickers to their in-group members with different preferences. In each trial, 7.8% of children distributed no sticker to their out-group members who shared their preferences.

Table 2. Number of children by number of stickers distributed to *same-preference* characters and trial in Tasks 1 and 2

stickers distributed to same-preference character	Task 1				Task 2			
	Trial 1	Trial 2	Trial 3	Trial 4	Trial 1	Trial 2	Trial 3	Trial 4
0	0	0	0	0	5	5	5	5
1	13	7	9	8	53	50	40	46
2	46	51	49	51	6	9	19	12
3	5	6	6	5	0	0	0	1

S3. Categories and Frequencies (in Percent) of Children's Justifications in Task 3

Following each evaluation of the degrees of goodness/badness of the character's behavior, the children were asked to explain their ratings. Explanation was coded into 17 categories (see Table 3). The coding scheme was adapted from Mulvey et al. (2014), which examined school-aged children's choices in conflict between shared norm and group membership. In addition to the categories used by Mulvey et al. (2014; 'fairness', 'group identity', and 'larger societal norm'), we used the themes of 'preference', 'possibility', 'reciprocity', 'mere description' to cover the responses that children gave in our study. Also, following Fu et al. (2016), we created coding categories based on the combination of the themes (e.g., group identity & preference) so that responses including more than one theme could be coded as a single category. Two independent raters coded all participants' responses to each justification question. Cohen's *K* was 0.98. Table 4 shows examples and frequencies (in percentage) of responses that fall into each of the 17 categories.

Table 3. Coding categories of children's justifications in Task 3

Category	Definition
Group identity	Responses that reference the group membership of the characters
Preference	Responses that reference the characters' preferences such as their favorite pets or toys
Group identity & preference	Responses that reference both the group membership and preferences
Possibility	Responses that judge the possibility of being friends or distributing resources
Normativity	Responses in which the child explicitly refers to a norm, making use of normative vocabulary (e.g., *should*, *must*, *ought to*, *supposed to*, *wrong*, *correct*, *normal*, and *more appropriate*)
Reciprocity	Responses in which the child explicitly refers to an expectation to be paid back at a later time
Fairness	Responses that reference the fairness of the division or liking

Group identity & possibility	Responses that reference both the group membership and possibility
Group identity & normativity	Responses that reference both the group membership and normativity
Group identity & reciprocity	Responses that reference both the group membership and reciprocity
Preference & possibility	Responses that reference both preferences and possibility
Preference & normativity	Responses that reference both preferences and normativity
Group identity & fairness	Responses that reference both the group membership and fairness
Group identity, preference, & normativity	Responses that reference both the group membership and preferences of the characters, together with normativity
Group identity, preference, & possibility	Responses that reference both the group membership and preferences of the characters, together with possibility
Mere description	Responses that only describe the numbers of resources given to the characters or the protagonist's friend choice
Unsure / nonsense	Not providing an answer. Or any statement that is uninterpretable, ambiguous, or irrelevant

Table 4. Examples and percentages of categories of children's justifications in Task 3 (*N* = 512)

Category	Percentage	Examples
Group identity	58.4	"Because these children are not in the same class"<br>"They are not even classmates"<br>"This child is the green class, but these children are the orange class"

Preference	10.2	"It's just a difference in what they like"<br>"They like different pets"<br>"Because this child likes the same toy as this child"
Group identity & preference	7.6	"They belong to different classes but play with the same toys"<br>"This child only considered pets and gave two stickers, but they are in different classes"
Possibility	0.2	"These children can be good friends"<br>"They cannot play together"
Normativity	2.7	"You should give your friends more"<br>"This child should be friends with this child"<br>"This child gave this child one sticker. It is wrong"<br>"This behavior is right"
Reciprocity	0.2	"If he gets along with this child, this child can help him, and he can help this child"
Fairness	1.8	"Because this child should draw a heart to both children"<br>"One must not draw a heart to only one child"<br>"Because it was not the same number of stickers"
Group identity & possibility	1.4	"These children cannot become good friends because they are not in the same classroom"<br>"Because they are not in the same class, they cannot play together. They can only greet"
Group identity & normativity	5.1	"Because children with the same class flag should play together, and one should not play with children from a different class with a different flag"
Group identity & reciprocity	0.6	"Otherwise, he won't help you. None of your classmates will help you"<br>"Because they are in the same class. These children can help each other"
Preference & possibility	0.2	"This child raises a snake, but this child can be friends with this child"

Preference & normativity	0.2	“This child likes the same toy as this child, and so should give this child two”
Group identity & fairness	0.2	“Because this child is not his class, and so he should give this child only one. Then everyone can have one sticker”
Group identity, preference, & normativity	0.6	“These children share favorite pets, but they are in different classes. So it does not look good that these children are friends”<br>“These children raise the same kind of pets, but a more important thing is that this child is the same class as this child. One ought to hang out with same-class peers”
Group identity, preference, & possibility	0.2	“This child is from a different class. They raise the same pets, but they cannot be friends”
Mere description	7.2	“This child (protagonist) likes this child more, and doesn’t like this child”<br>“Because this child likes to be friends with this child and does not like to be friends with this child”<br>“Because this child received more, and the other child received less”
Unsure / nonsense	3.2	“I don’t know”<br>“I like this toy, too”<br>“Because this child is polite”<br>“I like to be friends with my classmates”

References

Mulvey, K. L., Hitti, A., Rutland, A., Abrams, D., and Killen, M. (2014). Context differences in children's ingroup preferences. *Developmental Psychology*. *50*, 1507-1519. doi: 10.1037/a0035593

Fu, G., Luo, Y. C., Heyman, G. D., Wang, B., Cameron, C. A., and Lee, K. (2016). Moral evaluation of lying for one's own group. *Infant and Child Development*. *25,* 355-370. doi: 10.1002/icd.1941
